# The chirality origin of retinal-carotenoid complex in gloeobacter rhodopsin: a temperature-dependent excitonic coupling

**DOI:** 10.1038/s41598-020-70697-5

**Published:** 2020-08-19

**Authors:** Sankar Jana, Kwang-Hwan Jung, Mordechai Sheves

**Affiliations:** 1 Department of Organic Chemistry, Weizmann Institute of Science, 76100 Rehovot, Israel; 2 Department of Life Science and Institute of Biological Interfaces, Sogang University, Shinsu-Dong 1, Mapo-Gu, Seoul, 121-742 South Korea; 3 Present Address: School of Biology, Biomedical Science Research Complex, University of St Andrews, North Haugh, St Andrews, KY16 9ST UK

**Keywords:** Biophysical chemistry, Biophysical chemistry

## Abstract

Retinal proteins play significant roles in light-induced protons/ions transport across the cell membrane. A recent studied retinal protein, gloeobacter rhodopsin (gR), functions as a proton pump, and binds the carotenoid salinixanthin (sal) in addition to the retinal chromophore. We have studied the interactions between the two chromophores as reflected in the circular dichroism (CD) spectrum of gR complex. gR exhibits a weak CD spectrum but following binding of sal, it exhibits a significant enhancement of the CD bands. To examine the CD origin, we have substituted the retinal chromophore of gR by synthetic retinal analogues, and have concluded that the CD bands originated from excitonic interaction between sal and the retinal chromophore as well as the sal chirality induced by binding to the protein. Temperature increase significantly affected the CD spectra, due to vanishing of excitonic coupling. A similar phenomenon of excitonic interaction lose between chromophores was recently reported for a photosynthetic pigment-protein complex (Nature Commmun, 9, 2018, 99). We propose that the excitonic interaction in gR is weaker due to protein conformational alterations. The excitonic interaction is further diminished following reduction of the retinal protonated Schiff base double bond. Furthermore, the intact structure of the retinal ring is necessary for obtaining the excitonic interaction.

## Introduction

**Figure 1:**
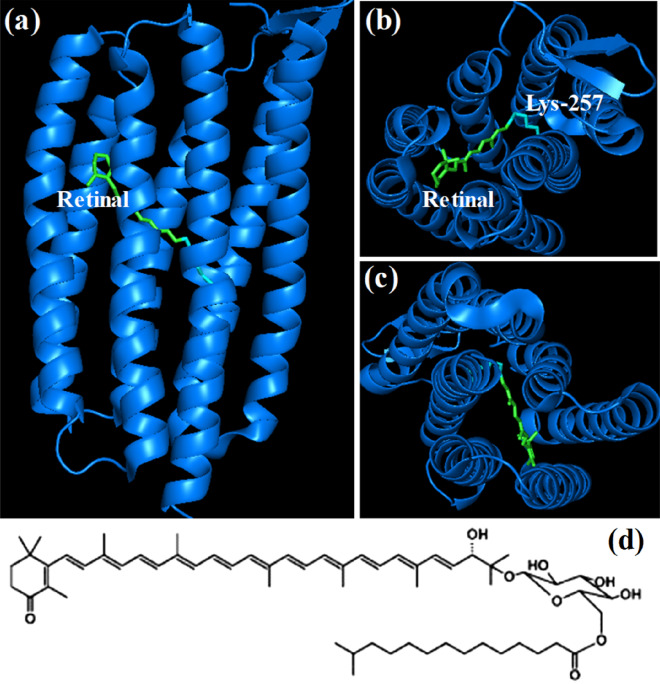
Secondary structure of gR protein was taken from the PDB (ID: 6NWD) file^15^. The retinal (green) and Lys-257 (cyan) are shown by stick model (**a**) Side view (**b**) Top view from N-terminus and (**c**) C-terminus end. (**d**) Chemical structure of the carotenoid sal.

Gloeobacter rhodopsin (gR) is a recently studied retinal protein which was found in thylakoid-less unicellular cyanobacterium Gloeobacter violaceus Pcc 7421^1,2^, heterogeneously expressed in *E. coli.* Similarly to other retinal proteins, it transfers protons from the cytoplasmic region to the extracellular region and acts as a light-activated proton pump^3,4^. Limited studies were carried out related to gR structure, function and spectral properties^1, 3,5–14^. gR is a type I rhodopsin in which the retinal chromophore is bound to Lys-257 amino acid residue of the seventh transmembrane G-helix by a protonated Schiff base formation (Fig. 1a–c)^10, 15^. It was revealed that the gR protein is capable of binding salinaxanthin carotenoid (sal, Fig. 1d) which transfers at least 40% of its absorbed energy to the retinal chromophore following light absorption^6^. gR has 50% residue identity and 42% sequence similarity to xanthorhodopsin (xR)^1^.

CD measurements have been widely used as a sensitive tool to investigate protein conformational changes, and to analyse chromophore-chromophore and chromophore-protein interactions in various proteins^16,17^. The CD spectrum of native xR, which contains two chromophores (retinal and sal), exhibits sharp positive bands at 513, 478, and 455 nm, and a negative band at 530 nm, which formed a biphasic shaped spectrum^18,19^. These CD bands arise only when the carotenoid sal is bound to the protein and forms the protein-carotenoid complex. Sal itself as an isolated chromophore has very weak CD bands in the visible region^20^. The CD bands of the complex vanished when the protein is denatured at high pH or the retinal protonated Schiff base linkage is cleaved following a reaction with hydroxylamine^19^. Different possibilities were proposed to explain the origin of the CD spectrum of xR. Balashov et al. proposed that mainly two factors contribute to the CD spectrum of xR. One is the excitonic coupling between the carotenoid and the retinal, and another is the induced chirality of the chromophores due to their binding to the protein^18,21^. CD excitonic coupling (EC) is originated from light excitation of two non-conjugated but energetically close chromophores, which their excited states are coupled, causing an energy splitting of the excited states into two different energy states. The transition to these states with different rotational strength generates a bisignate CD spectra (with opposite sign CD lobes) and observed Cotton effect (CE)^22^. We have proposed that the CD spectrum of xR originated from an excitonic coupling interaction between two sal molecules located in subunits of XR^19,23^, and from a retinal band. The CD of native bR is composed from bisignate curves of opposite signs at 593 nm, and at 531 nm. The cross-point is located at 560 nm close to the corresponding UV–Vis absorption maxima (557 nm). The biphasic CD spectrum of bR has been interpreted as a superposition of an intrinsic positive CD arising from the protein environment and the exciton coupling of the retinal chromophores within the bR trimer^24–26^. Alternatively, it was proposed that it is arising only due to superposition of the intrinsic CD spectra^27–29^.

Although the CD spectra of native bR, and xR-sal complex were widely studied, the exact origin of the CD spectra is not completely clear^18,19,21,23,24,27–32^. The CD spectra of native gR as well as in the presence of different types of carotenoids were studied previously, and it was proposed that the CD of the gR-carotenoid complex is due to induced chirality of the carotenoid^10,11^. pH-dependent oligomer-monomer transition, and their CD spectra were reported by Demura et al.^9^ Recently it was reported that the pentameric form of the pigment predominant at pH 8 while the monomeric form at pH 3^15,33^. However, in presence of sal, the exact origin of the observed positive and negative CD lobes with the enhancement of intensity and reversal of the CD band sign compared to the native gR, is not clear. Besides, it is also unclear how the CD spectral nature of gR and its complex with sal is affected by temperature and pH of the medium^9^.

In this work, we have examined the origin of the gR chirality, and the role that excitonic coupling and temperature play in affecting the CD spectra of gR, and its complex with sal. We propose that the CD spectrum of the gR-sal complex can be attributed mainly to the excitonic interaction between sal and the retinal chromophore. Temperature elevation affects the CD spectral shape and abolishes the excitonic coupling between the retinal and sal.

## Materials and methods

### Protein preparation, Sal extraction and purification

gR was grown and purified as described elsewhere^3,6,12^. Sal was extracted from cell membranes of *Salinibacter ruber*^23,34^. Following the addition of 10 mg/OD sodium dodecyl sulphate (SDS), the mixture was lyophilized overnight. Acetone was added and vortexed for five minutes to extract the sal chromophore, which was purified using a silica gel column, and eluted with 25:75 acetone: *n*-hexane and, finally, with pure acetone. Ethanol solutions of all-trans retinal, synthetic retinal analogues, and sal were incubated with Apo-gR to carry out the binding process. To avoid protein denaturation, the ethanol volume was kept less than 2% of the protein solution volume^6^. To maintain the required pH of Apo-gR samples, we used a 50 mM citrate/phosphate/Tris buffer in 0.06% DDM (*n*-dodecyl β-d-maltoside).

### Preparation of Apo-gR and artificial pigments

Apo-gR was prepared by incubating gR with 0.5 M freshly prepared hydroxylamine (pH 7.5) and irradiation for 20 min with a Schott 250 W cold light source (Carl Zeiss Microscopy, Jena, Germany) equipped with a heat-absorbing filter and an optic fibre (level 4B). The light was filtered through a long pass cut off filter (Schott, Mainz, Germany) with λ > 520 nm. The bleaching process was monitored by UV–Vis absorption spectral measurements. Next, the sample was filtered through a membrane filter (centricon of 10 000 MW) by centrifugation and washed with 0.02% DDM for 5–6 times to remove the retinal oxime and unreacted hydroxylamine. The sample was then stored at 4 °C to avoid reconstitution with all-trans retinal originating from residual retinal oxime^12^. The retinal analogues were synthesized as previously described^23,34–38^. Artificial pigments of gR were prepared by overnight incubation with Apo-gR (absorbance of 0.1–0.2 OD) with two equivalents of the synthetic retinal analogues (1.5 equivalents for all-trans retinal) in presence of 50 mM citrate/phosphate/Tris buffer (according to the required pH), and 300 mM NaCl in 0.06% DDM at 25 °C.

### CD spectral measurement

A Chirascan CD spectrometer (Applied Photophysics) was used for CD spectral measurements. All the CD spectra are given here in ellipticity θ in millidegree. A quartz cuvette of 10 mm path length was used for the measurements. The CD spectra were recorded with a 1 nm bandwidth resolution and in 1 nm steps at different temperatures using Peltier temperature controller. For temperature variation measurement, the sample was allowed to thermal equilibrate for 10 min at each temperature. The CD spectra were measured in the dark state using a 150 W air-cooled Xe arc lamp as light source and baselines were corrected by subtracting reference spectra of the corresponding buffer solution. We have checked that there are no photoexcitation during the CD measurement under dark state for the gR and its artificial pigments. During processing the CD spectra after internal reference correction (690–700 nm wavelength region was taken as a reference where no CD intensity was detected, except for 13-CF_3_ retinal analogue (Table 1) where we used 745–750 nm range), each spectrum was smoothed by 5 points using the adjacent averaging method. During the formation of the gR and artificial gR pigments in presence of sal, the CD spectra were recorded at 5 min interval for the first one hour followed by one-hour interval for the remainder of the experiment (i.e., overnight).

**Table 1:** Structure of all-trans and synthetic retinal analogues, and their CD and UV–Vis spectroscopic parameters.

Retinal no.	Retinal structure	Retinal Abs.	Abs. bands gR + sal		CD bands gR + sal	
			Pigment Abs.	Sal Abs.	Negative	Positive
1		555^a^	565	518, 486, 460	538^b^	514, 481, 456
2		586	590	518, 485, 456	548	514, 484, 453
3		633	632	520, 486, 456	580	518, 484, 456
4		580	585	520, 486, 456	543	515,482, 458
5		485	–	515, 484, 457	531	509, 477, 453
6		520	550	518, 484, 460	534	510, 478, 454
7		585	594	523, 490, 464	565	517, 486, 459

^a^Absorption maximum of gR at 555 nm when the retinal is all-trans retinal. ^b^Characteristic CD negative band. Values are in nm unit.

### UV–Vis spectral measurement

The UV–Vis spectral measurements were done using an Agilent 8453 diode-array spectrophotometer (Agilent Technologies, Palo Alto, CA) equipped with an Agilent 89090A thermostated cuvette holder in a 10 mm quartz cuvette at 25 °C in the dark state. The spectra were recorded after proper background correction with the corresponding buffer solutions using Tungsten/Deuterium lamp as the light source. The processed absorption and difference spectra were generated after internal reference correction (780–800 nm wavelength region was taken as a reference where no absorption was detected).

### Reduction of the protonated Schiff base double bond

Sodium borohydride (NaBH_4_) was added to the native gR or to the artificial pigments bound to sal to reduce the retinal-lysine protonated Schiff base (PBS) double bond^19^. The reduction process was carried out under illumination with a halogen lamp (12 V, 150 W), and the light was filtered through a long-pass cut off filter of λ_max_ > 520 nm (Schott, Mainz, Germany). Following the completion of the reduction process, the sample was dialyzed against 50 mM citrate buffer of pH 5, and 300 mM NaCl.

## Results

We have studied the CD and corresponding absorption spectroscopies of gR and its artificial pigments derived from synthetic retinal analogues following sal binding, to shed light on the origin of the CD spectra in the visible region, and its enhancement by sal binding. The binding of all trans retinal to apo-gR was studied previousely, and indicated high binding efficiency (ca. 85%)^12^. Similarly, the binding of retinal analogues to the apo-gR was studied. The binding of sal and other carotenoids to gR and CD spectra were studied by Balashov et al.^10,11^ and also indicated high efficiency of binding. We studied the effect of temperature on the CD Spectra. The CD bands and UV–Vis absorption maxima of gR pigments and its artificial pigments in their dark-adapted state are summarized in Table 1.

### pH and temperature effects on the CD spectra of gR

**Figure 2:**
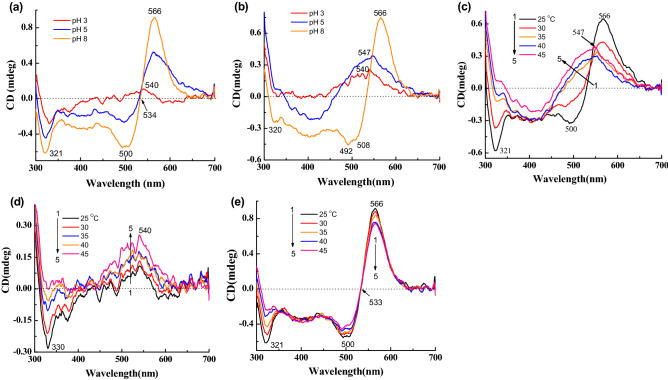
CD spectra of gR at pH 3, 5, 8 in 0.06% DDM, 300 mM NaCl (**a**) at 25 °C and (**b**) at 45 °C. CD spectra of gR at (**c**) pH 5 (**d**) pH 3 (**e**) pH 8, with variation of temperature from 25 to 45 °C in 0.06% DDM, 300 mM NaCl.

Figure 2a shows the CD spectra of gR at different pH values at 25 °C. At pH 3, gR has very low-intensity CD bands at 540 (+) and 330 nm (−), whereas, at pH 5, the CD spectrum is prominent with bands at 566 (+), 500 (−) and 321 nm (−). The intensity of the CD spectrum is further enhanced at pH 8^9^. Absorption spectra of gR at similar conditions^26^ (Fig. S1a,b), show pigment bands maxima at 546, 542 and 540 nm for pH 3, 5 and 8, respectively. Figure 2b shows the CD spectra of gR at pH 3, 5 and 8 at 45 °C temperature (gR CD spectral reversibility was observed until this temperature). Figure S1c,d represents the recorded absorption spectra at the same time and same condition as applied for CD measurement. Gradual temperature increase from 25 to 45 °C, indicates a shift of the CD band position from 566 to 547 nm at pH 5 (Fig. 2c), whereas, a slight change of CD intensity without any change of spectral band position at pH 3 and 8 was observed (Fig. 2d,e). Corresponding absorption and difference absorption spectra are presented in Fig. S2a–d.

### CD spectra of gR following binding of sal

**Figure 3:**
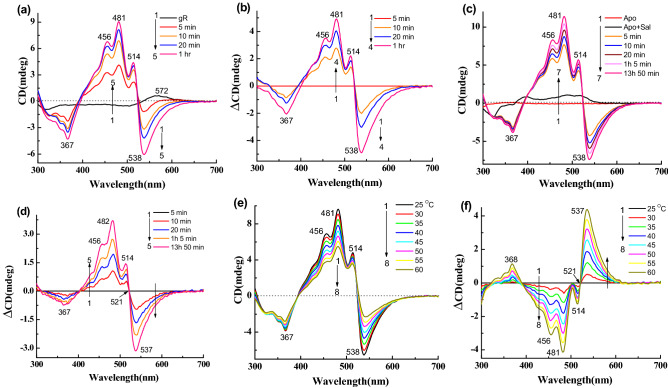
CD spectra of gR during incubation with sal, in 0.06% DDM containing 50 mM citrate buffer of pH 5 and 300 mM NaCl, (**a**) 1: gR, 2 → 5: spectra after addition of sal with different time intervals. (**b**) Difference spectra, 1 → 4: the spectrum obtained after 5 min. of sal addition was subtracted from others. (**c**) CD spectra during incubation of all-trans retinal with Apo-gR in presence of sal, 1: Apo-gR, 2: after addition of sal, 3 → 7: after addition of all-trans retinal with different time intervals. (**d**) Difference spectra, 1 → 5: the spectrum obtained after 5 min. of all-trans retinal addition was subtracted from each spectrum. (**e**) Temperature effect on the CD spectra of the gR-sal complex at pH 5, 1 → 8: spectra at different temperatures. (**f**) Difference spectra (spectrum obtained at 25 °C temperature was subtracted from others).

Figure 3a represents the CD spectra for reconstitution of gR with the carotenoid sal at 25 °C. gR itself has very weak CD spectrum at pH 5 (Fig. 2c) with bands at 566 (+), 500 (−) and 321 nm (−). Following binding of sal, negative CD bands were observed at 538, 367 nm and positive CD bands at 514, 481, 456 nm. The positive part of the CD spectrum has a similar spectral shape to that of sal absorption spectrum except for a small blue shift of the band positions (Table 1). The complex formation and the formation of the CD spectrum were almost completed within 1 h^12^. Fig. 3b represents the difference CD spectra obtained by subtraction of the spectra recorded after 5 min of sal addition from the other spectra. To check whether a similar complex is formed by reconstitution of Apo-gR and sal mixture with all-trans retinal (**1**), we monitored this process with CD spectroscopy at pH 5. As shown in Fig. 3c, the Apo-gR has almost no CD bands in the visible range, whereas, sal itself has a very weak CD spectrum in the presence of Apo-gR. Following reconstitution with the retinal chromophore, the CD spectrum of the complex was strongly enhanced, and yielded a negative band at 538 nm and a positive band at 481 nm. The difference spectra (Fig. 3d) show that the positive and negative bands intensities are similar. We have also monitored the absorption spectra recorded at similar conditions (Fig. S3a)^12^. We note that the CD spectrum of sal did not change after addition of the sal to the Apo-gR, (data not shown). We have further studied the temperature effect on the CD spectra of the gR-sal complex (Fig. 3e). Increasing the temperature was accompanied by a decrease of the CD bands intensities with a slight change of the positive band position, as demonstrated by the difference spectra (Fig. 3f). The difference spectrum is not identical to the original spectrum indicating that the original spectrum is composed of at least two components, in which only one is temperature dependent. We have calculated the temperature dependent component of the CD spectrum of gR-sal complex from the CD intensity at 538 nm due to intensity change by increasing the temperature from 25 to 60 °C which is approximately 65%. The corresponding absorption and difference absorption spectral changes are shown in Fig. S3b,c. It clearly indicates that sal experiences a conformational change, which is reflected in disappearance of a part of the vibrational fine structure at 486 and 518 nm absorption spectrum. Following cooling the system to 25 °C the CD spectrum regained its original bands, which indicates that the thermal process is reversible (Fig. S3d).

### CD spectra of artificial gR pigments in presence of sal

To further study the origin of the CD spectrum of gR, we have used artificial gR pigments derived from synthetic retinal analogues in which the native retinal ring and side-chain were modified. Consequently, the pigments absorption maxima were shifted, and the effect of these shifts on the CD spectrum was evaluated.

**Figure 4:**
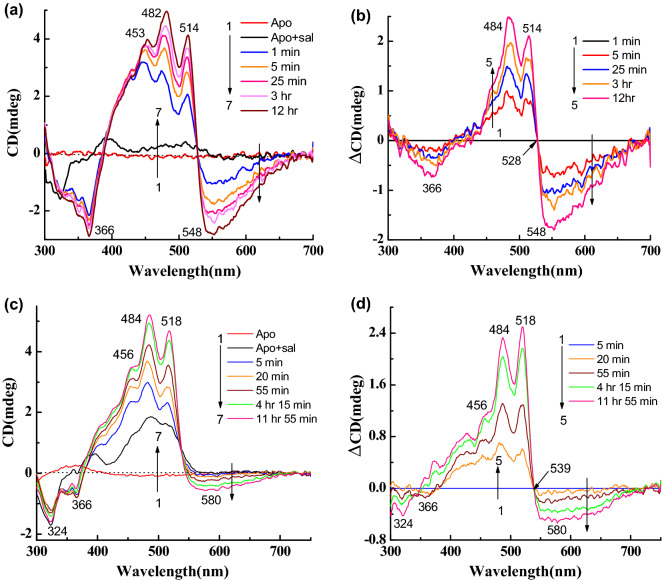
(**a**) CD spectra during reconstitution of 14-fluoro retinal analogue (**2**) with Apo-gR in presence of sal in 0.06% DDM containing 50 mM citrate buffer of pH 5 and 300 mM NaCl, (**a**) 1: Apo-gR, 2: after addition of sal, 3 → 7: after addition 14-fluoro retinal analogue with different time intervals. (**b**) Difference spectra, 1 → 5: the spectrum obtained after 1 min. of 14-fluoro retinal addition was subtracted from each spectrum. (**c**) Reconstitution of **13-CF**_**3**_ retinal analogue (**3**) with Apo-gR in presence of sal, 1: Apo-gR, 2: after addition of sal, 3 → 7: after addition **13-CF**_**3**_ retinal analogue (**3**) with different time intervals. (**d**) Difference spectra, 1 → 5: the spectrum obtained after 5 min. of **13-CF**_**3**_ retinal addition was subtracted from each spectrum.

First, we have incubated 14-fluoro (**2**) and 13-CF_3_ (**3**) retinal analogues with Apo-gR in the presence of sal to form the two artificial pigments (Table 1). As shown in Fig. 4a,b (difference spectra), the negative CD band of the artificial pigment derived from 14-fluoro retinal is at 548 nm which is 10 nm redshifted relative to the negative CD band of the gR-sal complex. The band is also wider than the CD band of the gR-sal complex, and the amplitude of the positive and the negative bands are not equal. To compare the spectral shape and band position of the negative band, the reconstituted final spectra of gR-sal complex and other synthetic retinal analogues are shown in Fig. S4a. For a better comparison of the redshifted negative band position, we have normalized the bands by dividing the spectra by the maximum negative amplitude (Fig. S4b). The artificial pigment derived from 13-CF_3_ retinal analogue (**3**) and its sal complex, has a wide negative CD band at 580 nm (Fig. 4c,d). The corresponding absorption spectra are shown in Fig. S5a-b. A temperature increase of the two gR artificial pigments induces a decrease of the CD bands intensities (Fig. S5c–f). The negative band almost disappeared at 55 °C but the positive band still has a significant intensity (Fig. S5c,e).

**Figure 5:**
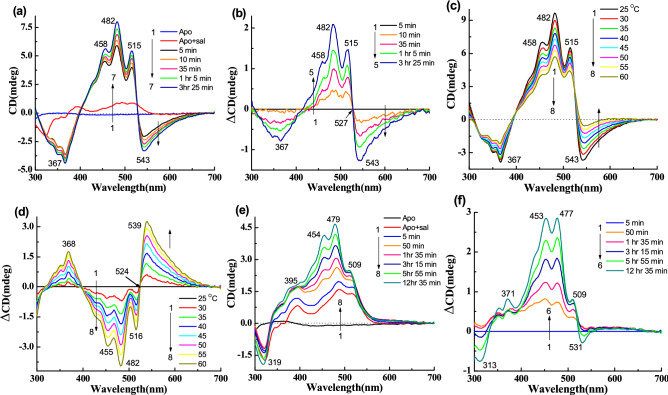
(**a**) CD spectra during reconstitution of retinal analogue **4** with Apo-gR in presence of sal in 0.06% DDM containing 50 mM citrate buffer of pH 5 and 300 mM NaCl, 1: CD spectra of Apo-gR, 2: after addition of sal, 3 → 7: CD spectra after addition retinal analogue **4** with different time intervals. (**b**) Difference spectra, 1 → 5: the spectrum obtained after 5 min. of retinal analogue **4** addition was subtracted from each spectrum. (**c**) Temperature effect on the CD spectra of the gR-**4**-sal complex at pH 5, 1 → 8: spectra at different temperatures. (**d**) Difference spectra (spectrum obtained at 25 °C was subtracted from others). (**e**) Reconstitution of retinal analogue **5** with Apo-gR in presence of sal, 1: Apo-gR, 2: after addition of sal, 3 → 8: after addition retinal analogue **5** with different time intervals. (**f**) Difference spectra, 1 → 6: the spectrum obtained after 5 min. of retinal analogue **5** addition was subtracted from each spectrum.

We have studied the CD spectra of artificial pigments derived from synthetic retinal analogues modified at the retinal ring. The retinal analogues included retinals with one additional ring double bond (**4**), without any ring double bond (**5**), without the retinal ring (**6**), and with aromatic core substituting the retinal ring (**7**) (Table 1). The CD and its difference spectra of retinal analogue **4** (Table 1) are shown in Fig. 5a,b. The 543 nm negative band is slightly redshifted (5 nm), but the other bands remain almost at the same positions compared to gR-sal complex. In addition, the negative CD band is wider than gR-sal complex (Fig. S4b). Here, the amplitude ratio between the positive and negative CD bands is 1.6. Following a gradual increase in temperature from 25 to 60 °C, both bands lost intensity at 60 °C temperature (Fig. 5c,d). The negative band at 543 nm almost disappeared whereas the positive band still had substantial intensity. This observation indicates that the original CD spectrum consists of at least two components. The absorption spectra obtained in similar conditions (Fig. S6a,b), clearly indicate almost no change of the original pigments at 60 °C temperature.

**Figure 6:**
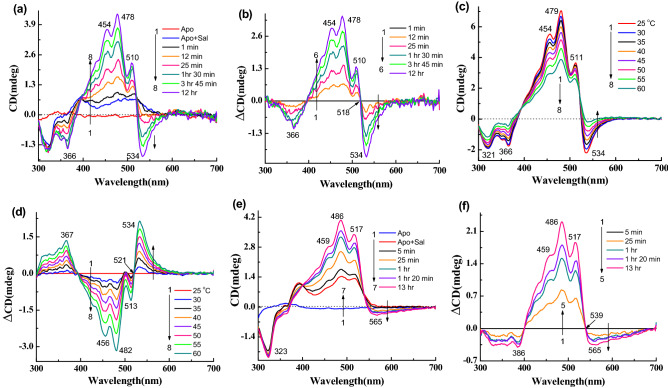
(**a**) CD spectra during reconstitution of linear retinal analogue **6** with Apo-gR in presence of sal in 0.06% DDM containing 50 mM citrate buffer of pH 5 and 300 mM NaCl, 1: CD spectra of Apo-gR, 2: after addition of sal, 3 → 8: CD spectra after addition linear retinal analogue **6** with different time intervals. (**b**) Difference spectra, 1 → 6: the spectrum obtained after 1 min. of retinal analogue **6** addition was subtracted from each spectrum. (**c**) Temperature effect on the CD spectra of the gR-**6**-sal complex at pH 5, 1 → 8: spectra at different temperatures. (**d**) Difference spectra (spectrum obtained at 25 °C was subtracted from others). (**e**) Reconstitution of retinal analogue **7** with Apo-gR in presence of sal, 1: Apo-gR, 2: after addition of sal, 3 → 7: after addition retinal analogue **7** with different time intervals. (**f**) Difference spectra, 1 → 5: the spectrum obtained after 5 min. of retinal analogue **7** addition was subtracted from each spectrum.

The CD spectra of the artificial pigment derived from retinal analogue **5**, which lacks the ring double bond (Table 1), show almost no negative CD band (Fig. 5e,f). A temperature increase led to an intensity decrease of the positive band and similar to gR-sal complex still the positive band maintains a significant intensity (Fig. S6c,d). The reversibility of the CD spectra was detected following increasing and decreasing temperature. The artificial pigment derived from retinal analogue **6** lacks the retinal ring but has an additional double bond instead of the retinal ring (Table 1). As shown in Fig. 6a,b a narrow negative CD band at 534 nm was observed accompanied by positive bands at 510, 478, 454 nm. Comparison with the CD spectra of gR-sal complex (Fig. S4b), indicates that the bands are slightly blue-shifted relative to the gR-sal complex. A temperature increase indicated similar results to that detected in the native gR-sal complex. We note that the negative CD band completely disappeared at 60 °C temperature (Fig. 6c,d), unlike the gR-sal complex.

Next, we used the aromatic substituted retinal analogue **7**, where the retinal ring is substituted by a *N*,*N* dimethyl amine aromatic ring (Table 1). The pigment derived from retinal analogue **7**, absorbs at 594 nm in the presence of sal^12^. The CD spectra of the artificial pigment **7** (Fig. 6e,f), exhibit a relatively weak broad CD negative band at around 565 nm and positive bands at 517, 486, 459 nm. The temperature effect on the CD spectra (Fig. S7a,b), showed similar results to the other artificial pigments.

### ***CD and absorption spectra following reduction with NaBH***_***4***_

**Figure 7:**
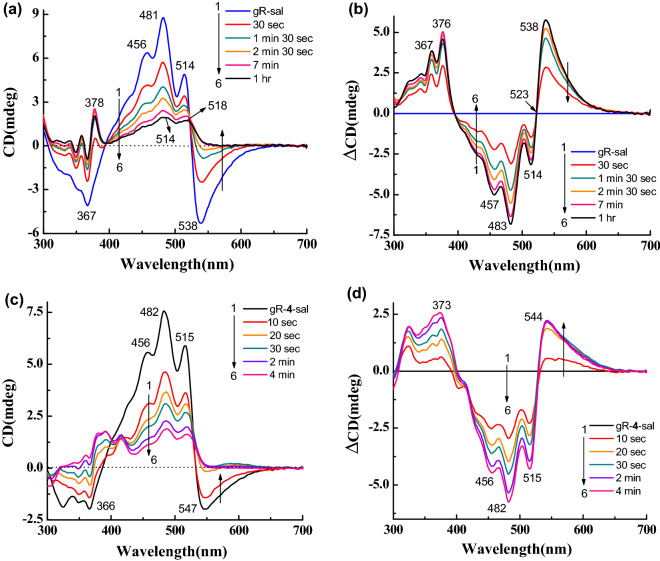
(**a**) CD spectra of gR-sal complex during reduction with NaBH_4_ in 0.06% DDM containing 300 mM NaCl, 1: CD spectrum of gR-sal complex, 2 → 6: CD spectra as function of irradiation time (**b**) Difference spectra, 1 → 6: the spectrum obtained before addition of NaBH_4_ was subtracted from each spectrum. For retinal analogue **4** (**c**) 1: CD spectra of gR-**4**-sal complex, 2 → 6: CD spectra during reduction as a function of irradiation time, (**d**) Difference spectra, 1 → 6: the spectrum obtained before the addition of NaBH_4_ is subtracted from each spectrum.

We reduced the protonated Schiff base double bond, which links the retinal chromophore to Lys-257 using NaBH_4_ to evaluate its effect on the CD spectra. As shown in Fig. 7a,b; the positive and negative CD bands of gR-sal complex almost vanished with a residual CD of sal which is similar to that observed in the presence of Apo-gR (Fig. S4a (for sal + Apo-gR)). The disappearance of the positive band is in contrast to the temperature effect on the gR-Sal complex CD spectrum in which the positive band maintained significant intensity even though the negative band almost disappeared. We also monitored the absorption spectra recorded during CD spectral measurements. The absorption and difference spectra (Fig. S7c,d), clearly represent the change mainly at the pigment band position with the loss of sal fine structure bands. The reduction experiment was performed as well with the artificial pigments derived from the synthetic retinal analogues. Reduction of the gR-**4**-sal complex led to complete disappearance of the negative CD band while a small intensity of the positive band remained probably due to the sal chromophore (Fig. 7c,d). The absorption and its difference spectra (Fig. S7e,f) show that except intensity decrease of the pigment absorption at 585 nm, sal bands decreased as well during the reduction process. The 13-CF_3_ retinal analogue (**3**) shows a complete loss of negative CD band and partial decrease of the positive CD band (Fig. S8a,b). The absorption and difference absorption spectra (Fig. S8c,d) clearly show the loss of the redshifted pigment band at 630 nm.

## Discussion

### The origin of gR CD spectrum and its pH and temperature dependence

**Figure 8:**
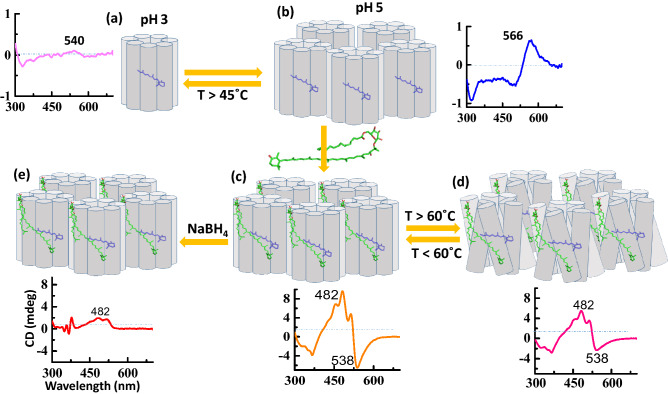
Schematic presentation of the effect of pH and temperature. The position and orientation of the chromophores and the protein are not known, and are indicated for illustration. (**a**) The monomeric form of gR at pH3; alpha helices are represented by cylinder and retinal by stick model. The CD spectrum is shown in the graph. (**b**) The pentameric form of gR at pH 5, and it’s CD spectrum. (**c**) The pentameric form of gR-sal complex and the possible retinal-sal interaction. CD spectrum at 25 °C is shown below. (**d**) gR-sal complex with CD spectrum at 60 °C temperature at which the excitonic coupling is lost possibly due to retinal protein 3D conformational changes. (**e**) Reduction of PBS double bond and loss of CD bands due to conformation change or/and blue shifted retinyl absorption.

The gR pigment contains only one chromophore (retinal) attached to Lys-257 by a protonated Schiff base double bond^1,8–12^. Therefore, the gR CD spectrum bands in the 300–700 nm region are attributed to the retinal induced chirality, and/or to excitonic interaction between retinal chromophores located in different proteins which are arranged in pentamers as previously reported^9,13,39^. At pH 5 and 8, it is evident that there are positive and negative CD bands at 566 and 500 nm, respectively, with Δε = 0 at 534 nm (Fig. 2a), which is close to the absorption maximum (540 nm) of wt-gR pigment^7,9,11^. Similar phenomenon was also reported for bR which has a negative band at 600 nm and positive band at 534 nm with Δε = 0 at 574 nm comparable with the wt-bR pigment absorption maximum at 567 nm^26^. The gR CD spectrum has opposite sign CD bands than bR and the first positive band is at 566 nm followed by a negative band at 500 nm^9^. Previously studied size-dependent chromatography proposed that at pH 3, gR adopts a monomeric form, whereas, at pH 8 only oligomers are present probably in the form of pentamers^9,15,33^. Our results clearly show that at pH 3, gR has a weak CD spectrum without characteristic positive and negative CD lobes at 25 °C (Fig. 2a) in keeping with monomer formation and lack of excitonic interaction between the retinal chromophores (Fig. 8a). The CD spectrum of gR at pH 5 was altered upon raising the temperature above 45 °C, and it resembled the CD spectrum at pH 3 and 25 °C. It may be explained by pKa alteration of the His87-Asp121 pair due to temperature increase. This pKa change can lead to pentamer to monomer transition probably by breaking the salt bridge, thereby effecting the CD spectrum due to vanishing of the excitonic interaction between the retinal chromophores (Fig. 8a,b)^9^. Therefore, the 547 nm CD spectral band of gR at pH 5 at 45 °C was similar to that observed at pH 3 at 25 °C (Fig. 2b). The CD spectrum was not altered at 45 °C at pH 8 even though the pKa of the His87-Asp121 pair was altered, still the protonation state of this pair was not changed since the pKa is still well below 8. Still, the possibility that the CD spectrum is altered following increasing the temperature to 45 °C at pH 5 due to protein conformation changes and not due to pKa change of the His87-Asp121 pair, cannot be completely excluded, but it is unlikely since at pH 8 the CD spectrum does not change at 45 °C.

### Chiral origin of the gR-sal complex and temperature-dependent excitonic coupling

The gR-sal complex exhibits much stronger CD spectrum than that of gR or sal in the presence of Apo-gR (Fig. 3a,c). The CD spectrum which is composed of negative and positive lobes, can be accounted for by several possibilities. The first possibility is based on excitonic interaction between sal and the retinal chromophore^21^. The second stems from the chiral conformation of the retinal in the presence of sal, while retinal-retinal excitonic coupling in a pigment pentamer form can be an additional cause for the CD spectrum^15,24,26,27^. Another possibility is based on a chiral conformation of sal in the presence of the retinal chromophore and nearby protein residues^19^. An excitonic coupling between two sal in the pentameric form cannot be excluded as well. A recent study suggested that excitonic interaction among chromophores in a photosynthetic protein strongly depends on temperature, and it is inversely affected by the temperature^39^. The present studies indicated that increasing the temperature of the gR-sal complex from 25 to 60 °C led to equally intensity decrease of the positive and the negative CD bands (Fig. 3f), which is completely reversible. We propose that these bands originated from excitonic interaction between the retinal and the sal chromophores. The excitonic interaction is lost at 60 °C possibly due to alteration of protein conformation, which may trigger sal and retinal conformational changes and change of the regular spatial organization, thereby eliminating the excitonic interaction. The intensity of both the negative and positive CD bands are reduced by warming to 60 °C, but still, a significant intensity of the positive band remains with a somewhat different shape relative to the original spectrum (Fig. 8c,d). We propose that the positive band is composed of an excitonic component of the interaction of sal with the retinal, as well as of a second sal component, which originates from the chirality of sal due to its specific chiral conformation in the protein, and/or the induced chirality by the protein. The absorption spectrum of the gR-Sal complex remains unchanged following temperature increase, which suggests that the complex is stable at 60^0^ C, and supports the suggestion that the CD spectrum is altered at 60^0^ C due to protein conformational changes and not protonation of the His87-Asp121 pair which is likely to affect the absorption maximum. Reduction of the PSB_,_ and disappearance of the induced CD (Fig. 7a,b), while detecting a residual CD band of sal, is in keeping with retinal-sal excitonic interaction in the retinal-sal complex (Fig. 8e). The presence of retinal-sal excitonic interaction may gain further support from the blue shift of the sal CD bands compared to the absorption band of the gR-sal complex (from 518, 486 and 460 to 514, 481, and 456 nm, respectively; Table 1). The blue shifts were observed as well in the CD spectra of the artificial pigments-sal complexes. Further support that the negative CD lobe is associated with the excitonic interaction between the retinal and the sal and not due to sal band, can be derived from the CD spectra of the artificial pigments. Substitution of the retinal by 14-fluoro retinal analogue (**2**) redshifts the negative band of the CD spectrum with decreasing intensity compared to gR-sal complex. Whereas in the case of 13-CF_3_ retinal analogue (**3**), the negative CD band is redshifted as well, with a significant decrease of intensity. The effect is probably due to the bulky group, which may affect the retinal conformation, thereby significantly decreases the excitonic interaction with the sal chromophore. Therefore, the redshifting, as well as the intensity decrease of the negative CD band, support as well the proposal that the negative band is associated with the retinal-sal excitonic interaction. The absorption maximum of the artificial pigment derived from retinal analogue **5** is significantly blueshifted (485 nm), and therefore the negative band of the CD spectrum which should be associated with the retinal analogue is possibly masked and neutralized by the opposite sign intense sal positive band (Fig. 5e,f). The protein-retinal ring interactions are important for imposing the appropriate retinal and sal conformation for efficient excitonic interaction. It can be concluded from the CD spectra of the artificial pigments derived from chromophores **6** and **7**. The linear chromophore **6** lacks part of the retinal ring while chromophore **7** has an aromatic core, which drastically alters the ring structure. While the CD spectrum of the artificial pigment derived from retinal analogue **6** still exhibits a negative band (somewhat weaker relative to native gR), the CD spectrum of the pigment derived from chromophore **7** lacks the negative band almost completely. It was proposed that sal ring carbonyl group is important for binding of sal to gR^10,11^. Our present studies indicate that retinal ring structure controls the specific retinal-sal interactions thereby affecting the excitonic interaction of the two chromophores. The CD spectra of the artificial pigments are affected less by the temperature increase relative to the native gR, since the excitonic interaction is weaker. Still, the positive band of sal is maintained at high temperature, indicating that the sal gains its chirality also in the artificial pigments and it is affected significantly by modifying the retinal structure.

Reduction of the PSB of the gR-sal complex with NaBH_4_ decreases much of the positive band and all the negative band CD intensities (Fig. 7a). Since the negative band is associated with the retinal chromophore it completely disappeared (Fig. 8e). The retinal absorption is significantly blueshifted to 357 nm, and therefore, the excitonic interaction between the two chromophores is abolished. Since the positive band lost significant intensity (beyond the excitonic interaction) we propose that the reduction of the Schiff base linkage affected the retinal conformation and thereby the conformation of the sal as well (Fig. 8e). We note that the absorption of the sal chromophore did not change following the protonated Schiff base reduction, which indicates that the sal conformation did not drastically change (Fig. S7c). The present results indicate that the possibility of the CD origin from excitonic interaction between two sal in a protein pentameric form is very unlikely. The shift of the CD negative band in the artificial pigments and its disappearance following the PSB reduction strongly indicates that the negative band is associated with the retinal chromophore (Figs 4d, 7c and S4b). We recently studied the origin of the CD spectra in xR^19^, but it appears that the situation in gR is completely different. We have proposed that the CD spectrum of xR mainly originated from the exciton interaction between two salalinixanthin chromophores located in different subunits. In addition, two contributions stem from the chiral conformation of the salalinixanthin within its binding site probably due the fixation of the 4-keto ring in a specific twisted conformation, and the contribution of the retinal chromophore to a negative lobe located at 550 nm. xR lacks a major excitonic interaction between the retinal and salalinixanthin probably due to unfavourable angle between the planes of retinal and salalinixanthin. The situation in gR is different and the CD of gR-sal complex has a component originated from excitonic interaction between retinal and salinixanthin which is possibly due to the favourable angle between the planes of retinal and salinixanthin. Unlike xR, gR-sal complex lacks excitonic interaction between the sal chromophores located at different gR monomers in the aggregated cluster possibly due to unfavoarble spatial arrangement required for such excitonic interaction. We have quantitatively estimated the components of the CD spectrum of gR-sal complex from the intensity of the CD spectra at 538 nm (CD intensity of gR-sal complex at 25 and 60 °C, Fig. 8c–e), which indicates that retinal-sal exciton contributes approximately 65% and remaining 35% from the sal chirality acquired by sal following binding to gR.

In summary, it can be concluded that the CD spectrum of gR originated from the excitonic coupling between retinal chromophores located in a protein pentameric form (Fig. 8b)^15,33^. Due to change of temperature, the His87-Asp121 pair arrangement is altered leading to pentamer to monomer transition and the corresponding change of CD spectra at pH 5 and vanishing of CD exciton^9^. In the presence of sal, the gR CD spectrum gains significant intensity. The spectrum is composed of two main contributions: (1) The chirality that sal acquires following retinal binding either by its specific conformation that may be associated with sal twisted ring-chain conformation, and/or induced chirality by the protein environment. (2) Excitonic coupling between sal and the retinal chromophore. Increasing the temperature to 60 °C eliminates the excitonic coupling, which may be due to conformational alteration.

## Supplementary information

Supplementary information

## Abbreviations

gR: Gloeobacter rhodopsin
Apo-gR/apo: Apo-protein of gR
Sal: Salinixanthin
CD: Circular Dichroism
xR: Xanthorhodopsin
bR: Bacteriorhodopsin
wt-bR: Wild type bacteriorhodopsin
DDM: *N*-Dodecyl β-d-maltoside
SDS: Sodium dodecyl sulphate
EC: Excitonic coupling
CE: Cotton effect
NaBH_4_: Sodium borohydride
PBS: Protonated Schiff base
