# Supplementary material for: The chirality origin of retinal-carotenoid complex in gloeobacter rhodopsin: a temperature-dependent excitonic coupling
Source: Sci Rep. 2020 Aug 19;10:13992. doi: 10.1038/s41598-020-70697-5 (PMC7438509; doi:10.1038/s41598-020-70697-5)
Supplement: Supplementary file 1 — Supplementary information [file 41598_2020_70697_MOESM1_ESM.docx]

*Supporting information for*

**The Chirality Origin of Retinal-Carotenoid complex in Gloeobacter Rhodopsin: A Temperature-Dependent Excitonic Coupling**

Sankar Jana^†^[^§^](https://pubs.acs.org/doi/full/10.1021/acs.jpcb.7b07523#notes-1)^*^, Kwang-Hwan Jung^‡^, [Mordechai Sheves](https://pubs.acs.org/author/Sheves%2C+Mordechai)^†^[^*^](https://pubs.acs.org/doi/full/10.1021/acs.jpcb.7b07523#cor1)

^†^Department of Organic Chemistry, Weizmann Institute of Science, Rehovot 76100, Israel.

^‡^Department of Life Science and Institute of Biological Interfaces, Sogang University, Shinsu-Dong 1, Mapo-Gu, Seoul 121-742, South Korea.

^*^Corresponding author, E-mail: sankjana@gmail.com. Tel: +44-1334-463401 Fax: +44-1334-462595.

^*^Corresponding author, E-mail: mudi.sheves@weizmann.ac.il. Tel: +972-8-9344320. Fax: +972-8-9343026.

^§^Author present address: School of Biology, Biomedical Science Research Complex, University of St Andrews, North Haugh, St Andrews, KY16 9ST, United Kingdom.

**Chemicals and Instruments**

N-dodecyl β-D-maltoside (DDM) was purchased from Chem-Impex Int'l Inc., Hydroxylamine hydrochloride from Sigma Aldrich. Sodium dodecyl sulfate (SDS), disodium hydrogen phosphate anhydrous, sodium dihydrogen phosphate monohydrate, and silica gel were purchased from Merck, USA. NaCl, NaOH, HCl, Acetone, n-hexane, tris buffer and citric acid anhydrous were purchased from Bio Lab Chemicals; Citric acid monohydrate from Fluka Chemicals and EtOH from J. T. Baker chemicals (Avantor). Double distilled water (DDW) was used for the preparation of all aqueous solution, and spectral graded solvents were used for spectral measurements. Sorvall RC 6 Plus Centrifuge- Thermo Scientific, Heraeus Multifuge 35-R centrifuge, Eppendorf centrifuge 5417R, Christ Alfa 1-4 Lyophilizer, Refrigerated Incubator Shaker with Fluorescent Lighting IS-971RF instruments were used during growing, extraction and purification of gR, xR and salinixanthin. PHM220 Lab pH Meter was used after proper calibration for the pH measurements.

**Figure S1.** Absorption spectra of gR at different pH values in 0.06% DDM, 300 mM NaCl (a) at 25 ºC and (b) normalized zoom out spectra to show the band shift (c) at 45 ºC and its (d) normalized zoom out spectra.

**Figure S2.** Absorption spectra of gR with variation of temperatures in 0.06% DDM, 300 mM NaCl (a) at pH 5 (b) difference absorption spectra, in which the spectrum at 25 ^o^C was subtracted from the corresponding spectrum (c) at pH 3 (d) at pH 8.

**Figure S3.** Absorption spectra in 0.06% DDM, 300 mM NaCl at pH 5. (a) Reconstitution of Apo-gR with all-trans retinal in presence of sal. (b) Temperature effect on the absorption spectra of gR**-**sal complex. 1→8: Spectra at different temperatures. (c) Difference spectra (spectrum obtained at 25 ºC temperature was subtracted from the corresponding spectrum). (d) CD spectra of gR-sal complex at different temperatures. 1→8: Spectra with decreasing temperature from 60 to 25 ºC with 5 ºC interval.

**Figure S4.** (a) The reconstituted CD spectra of all-trans retinal and synthetic retinal analogues. (b) CD spectra of all-trans retinal and synthetic retinal analogues gR-sal complexes following normalizing the negative CD band to -1. Scaling was done with respect to the red-sided negative CD band. Legend numbers indicate retinal analogue numbers presented in Table 1.

**Figure S5.** Absorption spectra in 0.06% DDM, 300 mM NaCl at pH 5. (a) Reconstitution of Apo-gR with **13-CF_3_** retinal analogue (**3**) in presence of sal. 1: Apo-gR. 2: After addition of sal. 3→7: After addition retinal analogue (**3**) with different time intervals. (b) Difference spectra. 1→5: Spectrum obtained after 5 min. of **13-CF_3_** retinal addition was subtracted from each spectrum. Temperature effect on the CD spectra of (c) Apo-**14-F**-sal complex at pH 5. 1→7: Spectra at different temperatures. (d) Difference spectra (spectrum obtained at 25 ºC was subtracted from the corresponding spectrum). (e) Apo-**13-CF_3_**-sal complex at pH 5. 1→7: Spectra at different temperatures. (f) Difference spectra.

**Figure S6.** Absorption spectra in 0.06% DDM, 300 mM NaCl at pH 5. (a) Temperature effects on the absorption spectra of gR-**4-**sal complex. 1→8: Spectra at different temperatures. (b) Difference spectra (spectrum obtained at 25 ºC was subtracted from the corresponding spectrum). (c) Temperature effect on the CD spectra of gR-**5-**sal complex at pH 5. 1→7: Spectra at different temperatures. (d) 1→7: Difference spectra.

**Figure S7.** Temperature effect on the CD spectra of (a) gR-**7-**sal complex. 1→6: Spectra at different temperatures. (b) 1→6: Difference spectra (spectrum obtained at 25 ºC was subtracted from each spectrum) in 0.06% DDM, 300 mM NaCl at pH 5. (c) Absorption spectra monitored during the reduction of gR-sal complex with NaBH_4_. 1: Spectrum of gR-sal complex. 2→5: Spectra during reduction at different irradiation times. (d) Difference spectra. 1→5: The spectrum obtained before addition of NaBH_4,_ was subtracted from each spectrum. (e) Reduction of gR-**4**-sal complex with NaBH_4_. 1: Absorption spectrum of gR-**4**-sal complex. 2→6: Absorption spectra during the reduction reaction at different irradiation times. (f) Difference spectra. 1→6: The spectrum obtained before addition of NaBH_4_ was subtracted from each spectrum.

**Figure S8.** (a) CD spectra of gR-**3**-sal complex during the reduction reaction with NaBH_4_ in 0.06% DDM and 300 mM NaCl. 1: gR-**3**-sal complex. 2→5: CD spectra during the reduction reaction at different irradiation times. (b) Difference spectra. 1→5: The spectrum obtained before addition of NaBH_4_ was subtracted from each spectrum. (c) Absorption spectra of gR-**3**-sal complex reduction with NaBH_4_. 1: gR-**3**-sal complex. 2→5: Spectra during the reduction at different irradiation times. (d) Difference spectra. 1→5: The spectrum obtained before addition of NaBH_4_ was subtracted from each spectrum.
